# No safety without agency: Trauma-informed public involvement

**DOI:** 10.1371/journal.pmen.0000530

**Published:** 2025-12-31

**Authors:** Elin Inge, Georgina Warner, Anna Pérez Aronsson

**Affiliations:** 1 CHAP, Department of Public Health and Caring Sciences, Uppsala University, Uppsala, Sweden; 2 Women’s Mental Health During the Reproductive Lifespan (WOMHER), Uppsala University, Uppsala, Sweden; Uppsala Universitet, SWEDEN

## No safety without agency: Trauma-informed public involvement

Public involvement in mental health research is meant to contribute to social change – but paternalistic approaches contribute to keeping the voices of those with lived experience of trauma at the margins. In this text, we will discuss how a trauma- and violence-informed approach could help researchers think about safety, which we will propose is a common “inhibitor”, preventing health researchers from embarking on public involvement. At the basis of our discussion is our work with co-researchers with experiences of forced migration and domestic violence and abuse (DVA).

Public involvement – conducting research together with representatives of the population the research is about (here termed ‘co-researchers’) – can improve research relevance, reduce research waste and support the democratization of research [1,2]. It is increasingly recognised in the health research community and encouraged by funders, and the mental health field is no exception. Public involvement can help reduce social exclusion and epistemic injustice; when under-represented groups are involved as co-researchers, they can bring rare and valuable experiential knowledge to research [3]. Yet, a known problem is that public involvement reproduces social structures; many co-researchers belong to the majority population, while people who have experienced multiple traumatic events and violence, such as forced migration and DVA, are rarely involved in mental health research [4]. This is both rooted in and reinforces social and epistemic injustices [3,5]. Low social participation and loneliness is common in forced migration [6], and intrinsic to DVA, as a consequence both of abuser’s isolation tactics and negative social responses [7].

While groups in marginalised positions are rarely involved as co-researchers, there is a sparking interest in the health research community. Many researchers are however hesitant and uncertain around methodology, ethical perspectives and practical feasibility.

## Safety concerns as an inhibitor to public involvement

Safety is often a first concern for researchers when considering public involvement. For instance, researchers may worry about risks such as re-traumatisation. This is a valid concern; involving co-researchers with experiences of trauma and violence entails various considerations. Yet, public involvement advocates argue that going too far to protect co-researchers risk excluding them from opportunities to make a difference for themselves and others, and potentially gain important skills and experiences. This has been referred to as the “participation vs protection” dilemma by Liabo et al. [8], who position this as a false dichotomy where researchers cannot make meaningful decisions about co-researchers’ safety without making decisions together with them. In addition, involvement processes could influence recovery and healing after trauma [9], which is another potential benefit that risk being lost if co-researchers are excluded from involvement.

## “Safe” - according to whom?

We propose that a trauma-informed approach could be helpful for researchers seeking to balance “participation vs protection”, by assisting considerations around how public involvement can be designed in a way that enhances healing rather than simply avoiding harm. An example of a trauma-informed approach to public involvement is Lamb et al.’s *Australian Framework for the ethical co-production of research and evaluation with victim survivors of domestic, family, and sexual violence* [10]. This framework introduces principles such as agency, transparency, healing informed, safety and support, among other principles. Regarding safety, the framework highlights that it is not up to researchers to decide on their own what is safe or not, but that this must be considered in a transparent process together with the co-researchers. If safety-related decisions are made by the researchers alone, co-researchers are denied agency and existing systems of epistemic injustice are reinforced. Instead, researchers need to support co-researchers to make those decisions and provide the necessary resources for them to participate in a safe way.

In a recent example from one of our projects, we applied the framework to plan our data collection together with a co-researcher with experience of violence. While the team saw value in the co-researcher conducting the data collection, we discussed risks of negative emotions when conducting a data collection linked to personal experiences. The co-researcher wanted to pursue data collection, but decided to take preparatory measures by joining a support group. We agreed to reflect continuously during the research process and be open to reconsidering.

## Towards a trauma-informed approach

For co-researchers with experiences of trauma and violence, public involvement can positively influence recovery and healing, and help reduce systems of epistemic injustice [9]. Public involvement can also have a transformative effect on mental health research, when based on trauma-informed principles. In our work with co-researchers with experiences of forced migration and DVA, we have seen clear examples of the transformative effects public involvement can have on mental health research – once we made active efforts to work with trauma-informed principles. When reflecting on the journey that our own research group has conducted since initiation our public involvement efforts, we see a clear development from a protection-oriented approach – where co-researchers, despite our good intentions, were unintentionally excluded from research decisions where their involvement would have been valuable – towards a transparent and mutual process, where decisions on who participates in which research activities are discussed and decided upon in collaboration with the whole team. While this is not a simple procedure, we continuously develop our approach based on trauma-informed principles.

In an attempt to collate and spread knowledge, we recently started the MARGIN network; a platform for researchers working with public involvement with groups exposed to trauma or violence. The network was initiated by Swedish researchers and professionals, as a space to share methods, experiences and good examples. The MARGIN network aim to share knowledge beyond the network, though seminars and resources, to support and strengthen researchers involving groups with experiences of trauma and violence, and through international collaborations. Through this network, we aim to contribute to discussions on how a trauma- and violence-informed approach could help researchers seeking to balance participation and protection, and conduct safe, meaningful and ethical public involvement in mental health research.
